# Elevated Serum Insulin-Like Growth Factor 1 Levels in Patients with Neurological Remission after Traumatic Spinal Cord Injury

**DOI:** 10.1371/journal.pone.0159764

**Published:** 2016-07-22

**Authors:** Arash Moghaddam, André Sperl, Raban Heller, Kevin Kunzmann, Viola Graeser, Michael Akbar, Hans Jürgen Gerner, Bahram Biglari

**Affiliations:** 1 Heidelberg Trauma Research Group, Department of Trauma and Reconstructive Surgery, Center for Orthopedics, Trauma Surgery and Spinal Cord Injury, Heidelberg University Hospital, Heidelberg, Germany; 2 Institute for Medical Biometry and Informatics, Heidelberg University Hospital, Heidelberg, Germany; 3 BG Trauma Centre Ludwigshafen, Department of Paraplegiology, Ludwigshafen, Germany; Hertie Institute for Clinical Brain Research, University of Tuebingen., GERMANY

## Abstract

After traumatic spinal cord injury, an acute phase triggered by trauma is followed by a subacute phase involving inflammatory processes. We previously demonstrated that peripheral serum cytokine expression changes depend on neurological outcome after spinal cord injury. In a subsequent intermediate phase, repair and remodeling takes place under the mediation of growth factors such as Insulin-like Growth Factor 1 (IGF-1). IGF-1 is a promising growth factor which is thought to act as a neuroprotective agent. Since previous findings were taken from animal studies, our aim was to investigate this hypothesis in humans based on peripheral blood serum. Forty-five patients after traumatic spinal cord injury were investigated over a period of three months after trauma. Blood samples were taken according to a fixed schema and IGF-1 levels were determined. Clinical data including AIS scores at admission to the hospital and at discharge were collected and compared with IGF-1 levels. In our study, we could observe distinct patterns in the expression of IGF-1 in peripheral blood serum after traumatic spinal cord injury regardless of the degree of plegia. All patients showed a marked increase of levels seven days after injury. IGF-1 serum levels were significantly different from initial measurements at four and nine hours and seven and 14 days after injury, as well as one, two and three months after injury. We did not detect a significant correlation between fracture and the IGF-1 serum level nor between the quantity of operations performed after trauma and the IGF-1 serum level. Patients with clinically documented neurological remission showed consistently higher IGF-1 levels than patients without neurological remission. This data could be the base for the establishment of animal models for further and much needed research in the field of spinal cord injury.

## Introduction

Spinal cord injury continues to be one of the biggest challenges in modern medicine. Even though promising approaches have been investigated recently[1–3], we have not yet succeeded in creating an adequate therapy concept that promises healing or at least partial remission by reintroducing integrity to lesioned nerve pathways and thereby restoring their function [4, 5]. Injury to the spinal cord leads to loss of sensory, motor or autonomic nerve function and ranges from partial impairment of individual modalities in mild cases up to total loss of all function in cases of complete disruption of the spinal cord [4, 6–9]. After life-threatening injuries have been addressed in the hyper-acute phase, acute and subacute treatment is primarily used to prevent further progression of the primary injury and to reduce further loss of neurological function [5, 6]. The preservation of residual function in patients after spinal cord injury may mean the difference between total dependency and loss of quality of life, or possibly a reintegration in their professional as well as private activities and improved recovery after spinal cord injury [10, 11]. The impact on the quality of the patient’s life is evident [8]. Stopping the progression of neurological damage with early relief operations and the best supportive care possible as well as physical therapy and exercise administered early in hospitalization to regain old skills is of great importance in the treatment of traumatic spinal cord injury. Recent investigations try to reveal additional ways to enforce neuroprotection and therefore prevent further loss of nerve tissue [4, 12]. After the acute phase, which includes the trauma itself, the laceration or contusion of neurons and the surrounding glia, vessels and connective tissue, a second, subacute phase, in which the inflammatory process is set in motion, is further damaging to tissue but also for introducing repair mechanisms [13].

A major problem is the lack of sufficient and evidence based therapeutic concepts for the treatment of acute spinal cord injury [14]. The 2013 Update for Guidelines for the Management of Acute Cervical Spine and Spinal Cord Injuries [15] rated 77 out of 112 recommendations as Class III (case series and expert opinion) due to missing evidence and an inappropriate study design for higher classified recommendations. In 2002, Kwon et al reviewed the literature for the quality of animal studies in spinal cord injury and could confirm their important role even though the transferability of findings in animal models to humans remains unclear[16]. They also pointed out how important it is to establish suitable animal models depending on the purpose of the study. In previous studies we could demonstrate, that it is possible to verify biochemical processes after traumatic spinal cord injury depending on the neurological outcome [17].

In our study we investigated the serum levels of IGF-1 in patients after traumatic spinal cord injury over a three-month period. Insulin-like growth factor 1 (IGF-1) or somatomedin C (SM-C) is a 70 amino acid long growth factor existing in various isoforms. It is detectable in blood serum. Most IGF-1 is made in the liver and is soluble. But the expression of IGF-1 could also be detected in muscle cells, bone cells, chondrocytes and presumably in a variety of other body cells with most paracrine effects [18].

In 2009, Hollis et al performed a C3 dorsal column transection in 344 adult Fischer rats and installed IGF-1 secreting marrow-stem cells via a graft put into the lesion cavity. They found a 93% corticospinal motor neuron cell survival rate compared to 49% in controls [19]. In another study, IGF-1 was shown to be able to prevent motor neuron excitotoxicity after exposure to elevated glutamate levels in vitro. Even up to 4 weeks after motor neuron injury, significantly higher survival of cells was detected after IGF-1 had been administered [20]. Vincent et al came to similar results and additionally stressed the importance of the right timing for the commencement of treatment [21].

The aim of our study was to investigate whether significant changes in IGF-1 levels can be observed in the acute, subacute and intermediate phases after traumatic spinal cord injury. Furthermore, we searched for correlations between our measurements and patient outcome.

## Material and Methods

Between 2010 and 2015, data of 45 patients (35 male, 10 female) after traumatic spinal cord injury was obtained in the Berufsgenossenschaftliche Unfallklinik Ludwigshafen (BG Trauma Centre). Venous blood was taken using a fixed protocol at 11 different time-points after trauma: At admission (on average less than one hour after trauma), four, nine, 12, 24 hours, three, seven days, two, four, eight, and 12 weeks after admission. The blood samples protocol is shown in Table 1. Missing samples in early time-points (0 hours to 1 day after injury) were mostly due to highly urgent procedures such as trauma surgery and intensive care. Missing samples in late time-points of the protocol were more likely due to failed collection of samples or loss to follow-up. For an overview of the number of analyzed samples at each time-point see S1 Fig. After 20 minutes of coagulation, the blood was centrifuged at 3000rmp, aliquoted and stored at—80 ° Celsius until analysis. The patient data are shown in Table 2. In order to systematically document the neurological damage in accordance with the *International Standards for Neurological Classification of Spinal Cord Injury* (ISNCSCI), the ASIA (American Spinal Injury Association) Impairment Scale (AIS) was used. The evaluation was conducted with responsive and cooperative patients and standardized by specially trained personnel at admission and discharge. IGF-I ELISA (R&D Systems, Minneapolis, MN) was used to determine levels of IGF-1 in venous blood serum. Mean IGF-1 levels at all measured time-points are shown for all subpopulations in S2 Fig. Additionally, the major subpopulations (all Patients/G1/G2/all AIS A/all AIS B-D) are displayed as boxplots and whiskers for each individual time-point in S3 Fig. Quantitative analysis of serum samples was carried out strictly according to the manufacturer’s instructions. In order to reduce measurement errors, all IGF-1 serum concentrations were measured in duplicate determinations. The performing laboratory staff was blinded by use of pseudonyms to all patient data, including AIS scores. The ELISA analysis was conducted in a S3 laboratory of the University Heidelberg in accordance with § 7 of the Genetic Engineering Act (GenTG). There was a positive vote of the ethics committee Rheinland Pfalz (no. 837.266.09) and an approval by the local Ethics Committee of the University of Heidelberg (S-514/2011) for the current study “*Elevated Serum Insulin-like Growth Factor 1 Levels in Patients with Neurological Remission after Traumatic Spinal Cord Injury*”. It is registered under the title “Cytokin Expressionsmuster von Patienten mit traumatischer Rückenmarksverletzung” (Study-ID: DRKS00009917/ Date of Registration: 23.03.2016/ Universal Trial Number (UTN): U1111-1179-1620) at the German Clinical Trial Register (*Deutsches Register Klinischer Studien–DRKS)*. Data management was performed according to good scientific practice. All study participants signed and dated a consent form willingly and could voluntarily choose to leave the study at any time and for any reason. Exclusion criteria were the following: non-traumatic spinal cord injury, traumatic brain injury (TBI), severe abdominal trauma, traumatic amputation of extremities and coma. Participants were not given methylprednisolone sodium succinate (MPSS) during study participation.

**Table 1:** Blood sample protocol. Numbered list of blood sample collection with corresponding time-points.

Blood samples protocol	
Number	Time-points
1	0 hours
2	4 hours
3	9 hours
4	12 hours
5	1 day
6	3 days
7	7 days
8	14 days
9	1 month
10	2 months
11	3 months

**Table 2:** Demographic and clinical characteristics of subjects. Abbreviations: NLI = Neurological Level of Injury; AO = AO-Classification; AIS = ASIA (American Spinal Injury Association) Impairment Scale; Paralysis: cP = complete Paraplegia, iC = incomplete Paraplegia, cT = complete Tetraplegia, iT = incomplete Tetraplegia; GCS = Glasgow Coma Scale. Age is expressed as mean years ± standard deviation. Neurological remission was defined as improvement in AIS. Four patients had posttraumatic spinal cord injury (SCI) without vertebral fractures.

			Gender		Etiology			AO				NLI			Initial AIS				Final AIS				Paralysis				GCSØ
Patients	Number	Age (years)	♂	♀	Fall	Traffic	other	A	B	C	-	C	Th	L	A	B	C	D	A	B	C	D	cP	iC	cT	iT	
**All Patients**	45	42,36 ± 19,07	35	10	31	12	2	23	13	5	4	20	14	11	22	9	13	1	15	3	12	15	12	14	5	14	13,93
**G1 (Remission)**	26	40,23 ± 19,78	18	8	20	6	0	14	7	2	3	9	8	9	7	8	11	0	0	2	10	14	6	12	0	8	14,33
**G2 (no Remission)**	19	45,26 ± 18,17	17	2	11	6	2	9	6	3	1	11	6	2	15	1	2	1	15	1	2	1	6	2	5	6	13,42

### Statistical Analysis

All statistical calculations were performed either with SPSS (IBM SPSS Statistics version 21.0) or R version 3.2.3 [22] using the package “pROC” [23] for ROC analysis. Correlation analyses were conducted between all variables of interest. In order to choose the best fitting statistical analysis, we tested the data of each individual time point for normality, using both, the Kolmogorov-Smirnov-Test and the Shapiro-Wilk-Test. Because of inconsistent test results, weather our data is normally distributed or not, and the relatively small number of cases (*n* = 45), we decided to use non-parametric tests. For test results see S2 Table. To detect location shifts between groups (comparison of IGF-1 levels in different subpopulations at certain time-points) the non-parametric Mann-Whitney U-Test for independent samples was used. To determine location shifts between IGF-1 levels within one group at different time-points the Wilcoxon Signed Rank Test for dependent samples was used. The Chi-square test was used to assess possible statistically significant differences in nominal data: Sex, Etiology of Accident, AO-Classification, Neurological Level of Injury, the type of paralysis and GCS.

Comparison of more than two independent samples were conducted using the Kruskal-Wallis test. Binary logistic regression was employed to assess the predictive power of IGF-1 for an improvement in AIS score while adjusting for potential covariates. Model selection for the logistic regression was based on AIC (Akaike Information Criterion). The best model fit with respect to AIC and including due to statistical as well as clinical relevance important variables and IGF-1 was achieved by the co-variables paralysis, neurological level of injury, mean IGF-1 serum level of the first 24 hours, initial AIS score and age. Besides model fit measured by AIC, clinical relevance was taken into account in the modelling process by requiring IGF-1 and age to be always part of the model [24] as IGF-1 is the main focus of this study and age due to the fact that it correlates to IGF-1[25]. Inclusion of any clinically justifiable interaction effects for example NLI and paralysis as well as age and mean IGF-1 levels of the first 24 hours resulted in deterioration of AIC and were thus not included in the final model. The dependent variable is remission / no remission (G1/G2). The corresponding point estimates, 95% confidence intervals and p-values for the adjusted odds ratios are given in Table 3. All statistical analysis and p-values are unadjusted for multiple testing and should be interpreted descriptively. Further studies with an elevated sample size are required in order to perform generalized estimating equations in a more expansive way including more variables. The results might give us an idea of the specific influence of each variable regarding the respective criterion. Furthermore, possible main effects and potential interaction terms might be detected.

**Table 3:** Odds ratios, CIs’ and p-values of variables included the preferred logistic regression models I (Table 3A) and II (Table 3B). Abbreviations: Paralysis: cP = complete Paraplegia, iC = incomplete Paraplegia, cT = complete Tetraplegia, iT = incomplete Tetraplegia; NLI = Neurological Level of Injury; MV = mean IGF-1 serum levels of the first 24 h; A_DB = dichotomized initial AIS = ASIA (American Spinal Injury Association) Impairment Scale, 0 = A, 1 = B-D; AGE = age of the subjects.

**A**				
Model I			**Confidence Interval**	
**Variables**	**p**	**Odds ratio**	**2.50%**	**97.50%**
**Intercept**	0.39	0.02	1.83e-06	67.79
**Paralysis**	0.19	3.44	0.69	32.51
**NLI**	0.51	1.98	0.30	19.73
**MV**	0.60	1.01	0.98	1.04
**A_DB**	0.02	0.16	0.03	0.73
**AGE**	0.61	1.01	0.96	1.07
**B**				
Model II			**Confidence Interval**	
**Variables**	**p**	**Odds ratio**	**2.50%**	**97.50%**
**Intercept**	0.39	2.34	0.37	18.84
**MV**	0.22	0.99	0.97	1.01

The predictive performance of the logistic regression model was analyzed using the receiver-operating characteristic (ROC) curve.

### Definition

Neurological Remission: Neurological Remission was defined as an improvement in the AIS score ≥ 1 from the initially determined grade at admission to the AIS score at discharge from the hospital.

## Results

### Patient Demographics

(see Table 2)

The entire collective consisted of 45 patients, including 35 males and 10 females. The collective was divided into two major subpopulations. Group one (G1) included all patients with neurological remission. Group two (G2) consisted of all patients without neurological remission. These two major subpopulations were further divided into their affiliation in either AIS A (both with and without remission; seven AIS A↑ and 15 AIS A↔) or AIS B-D (both with and without remission; 19 AIS B-D↑ and 4 AIS B-D↔). In the entire collective the average age was 42.36 ± 19.07 years. The etiology leading to the spinal cord injury was composed as follows: 31 falls (68.9%), twelve traffic (26.7%) and two others (4.4%). The neurological levels of injury (NLI) were all between the cervical spinal cord on the neurological level C3 and the lumbar spinal cord on the neurological level L4. 20 cervical (44.4%), 14 thoracic (31.1%) and 11 (24.4%) lumbar lesions. The initial AIS scores consisted of 22 A (48.9%), nine B (20.0%), 13 C (28.9%) and one D (2.2%) classified injuries. In the final classification by AIS there were 15 A (33.3%), three B (6.7%), 12 C (26.7%), and 15 D (33.3%) scores. The types of paralysis were divided into twelve (26.7%) patients with complete paraplegia, 14 (31.1%) with incomplete paraplegia, five (11.1%) with complete tetraplegia and 14 (31.1%) with incomplete tetraplegia. Group G1 (18 male, 8 female) with 40.23 ± 19.78 years was younger on average than group G2 (17 male, 2 female) with 45.26 ± 18.16 years on average. Group G1 showed 20 falls and four traffic related accidents leading to SCI. Initial AIS scores were distributed as follows: seven AIS A, eight AIS B and eleven AIS C. In the distribution of the final AIS scores there were two AIS B, ten AIS C and 14 AIS D graded patients. In G2, the aetiology of accidents leading to SCI consisted of eleven falls, six traffic related and two other causes. Initial AIS scores were 15 AIS A, one AIS B, two AIS C and one AIS D. The clinical characteristics of the patients are summarized in Table 2. No statistically significant association in nominal data for Sex, Etiology of Accident, AO-Classification, Neurological Level of Injury, the type of paralysis nor GCS could be determined (*p* > 0.05; Chi^2^-Test was used to test each combination of nominal data for significant association among themselves; data not shown).

### Analysis

#### Entire collective

**Fig 1:**
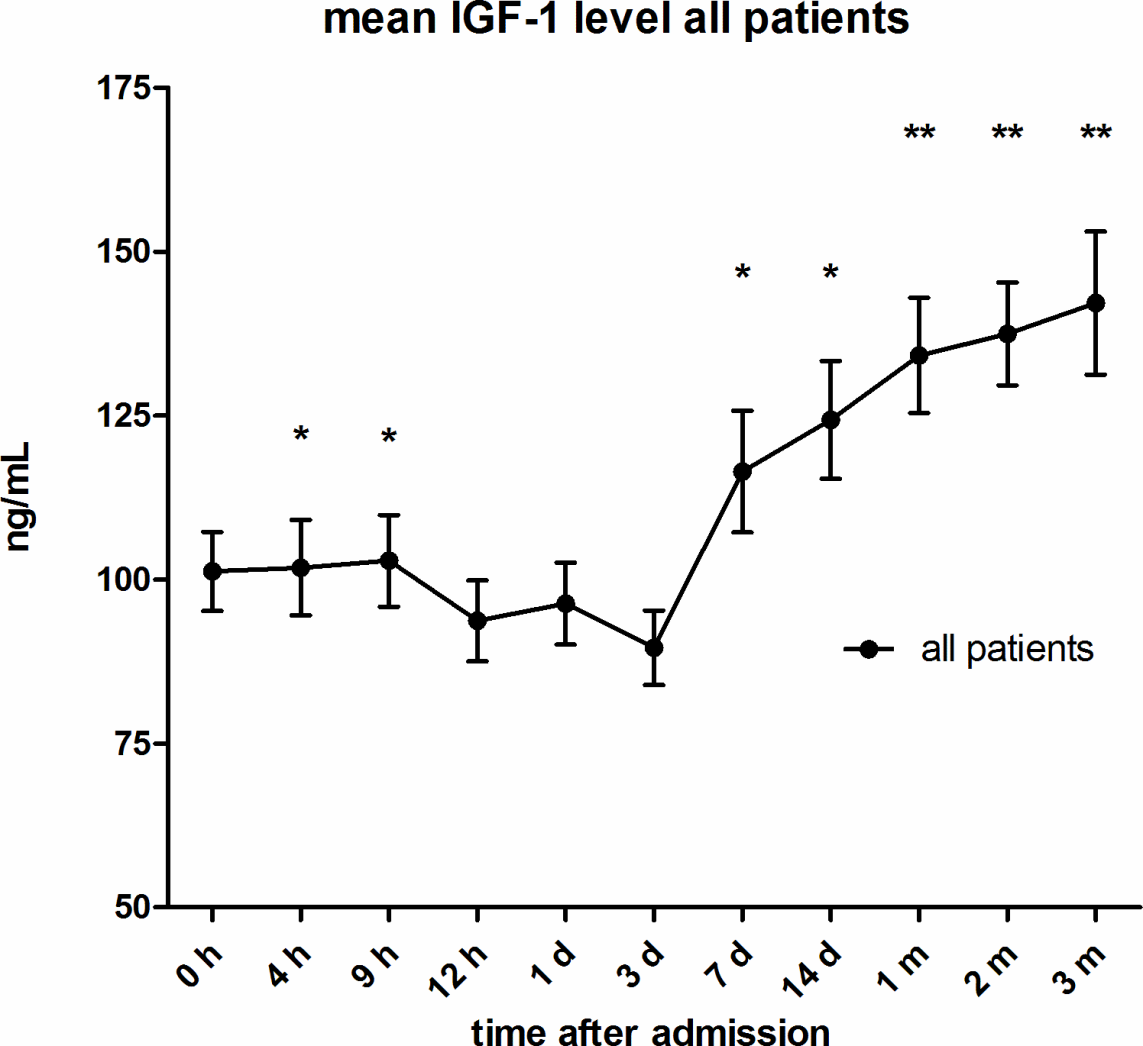
Mean IGF-1 serum levels of all traumatic SCI patients. During observation period of 3 months after injury, expressed as mean values ± standard error of the mean. The Wilcoxon signed-rank test assessed significant differences from the admission level (0h), *p < 0.05, **p < 0.001.

**Fig 2:**
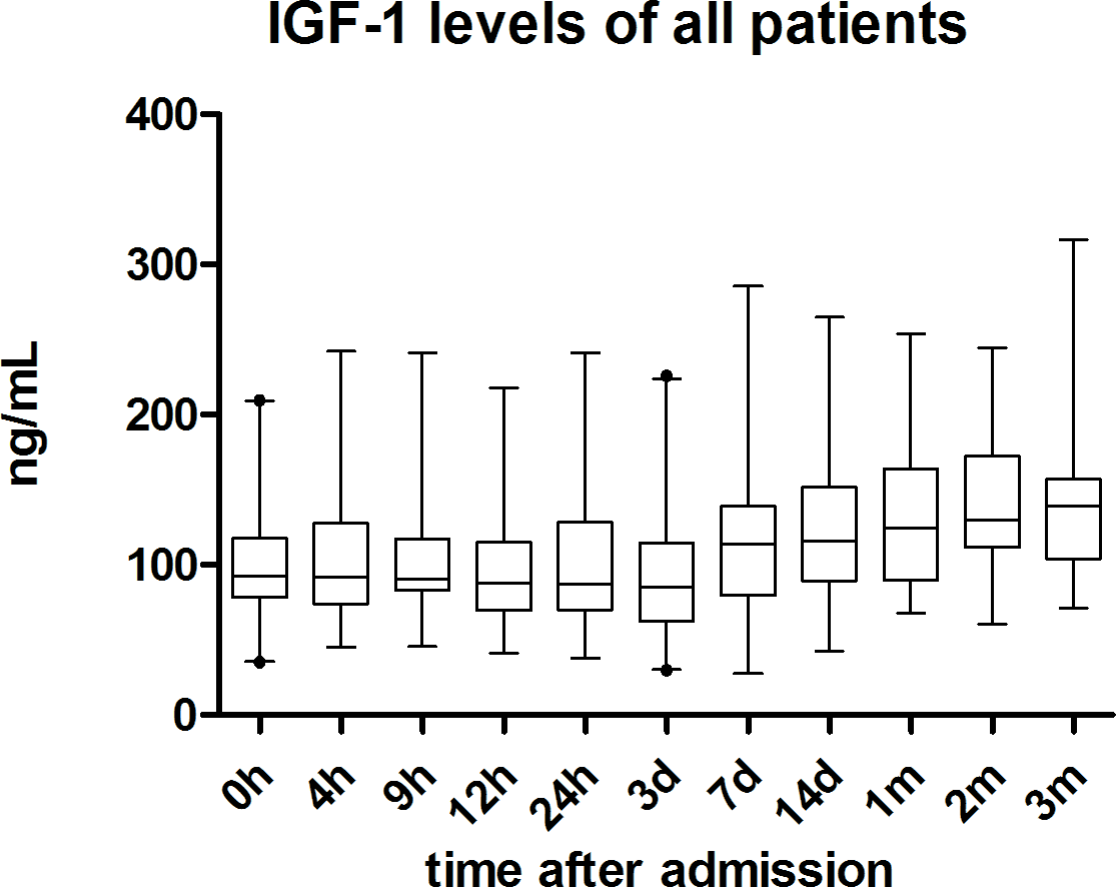
IGF-1 serum level Boxplots and Whiskers of all traumatic SCI patients. IGF-1 serum levels during 3-month observation period are displayed as boxplots and whiskers. Whiskers are displayed as 97,5 (upper whisker) and 2,5 (lower whisker) confidence intervals. Dots are indicating outliers.

(see Figs 1 and 2) IGF-1 serum levels were significantly different from initial measures four, nine, hours, seven, 14 days after injury (p < 0.05; Wilcoxon signed-rank Test) as well as one, two and three months after injury (p < 0.001; Wilcoxon signed-rank Test). Twelve hours after injury levels dropped from initially measured (at admission) 101.26 ng/mL to 93.70 ng/mL. IGF-1 levels eventually rose significantly seven days after trauma to 116.47 ng/mL and continued to rise but with a decreasing slope until the end of the period of observation to 142.20ng/mL.

#### Comparison of Group 1 and Group 2

**Fig 3:**
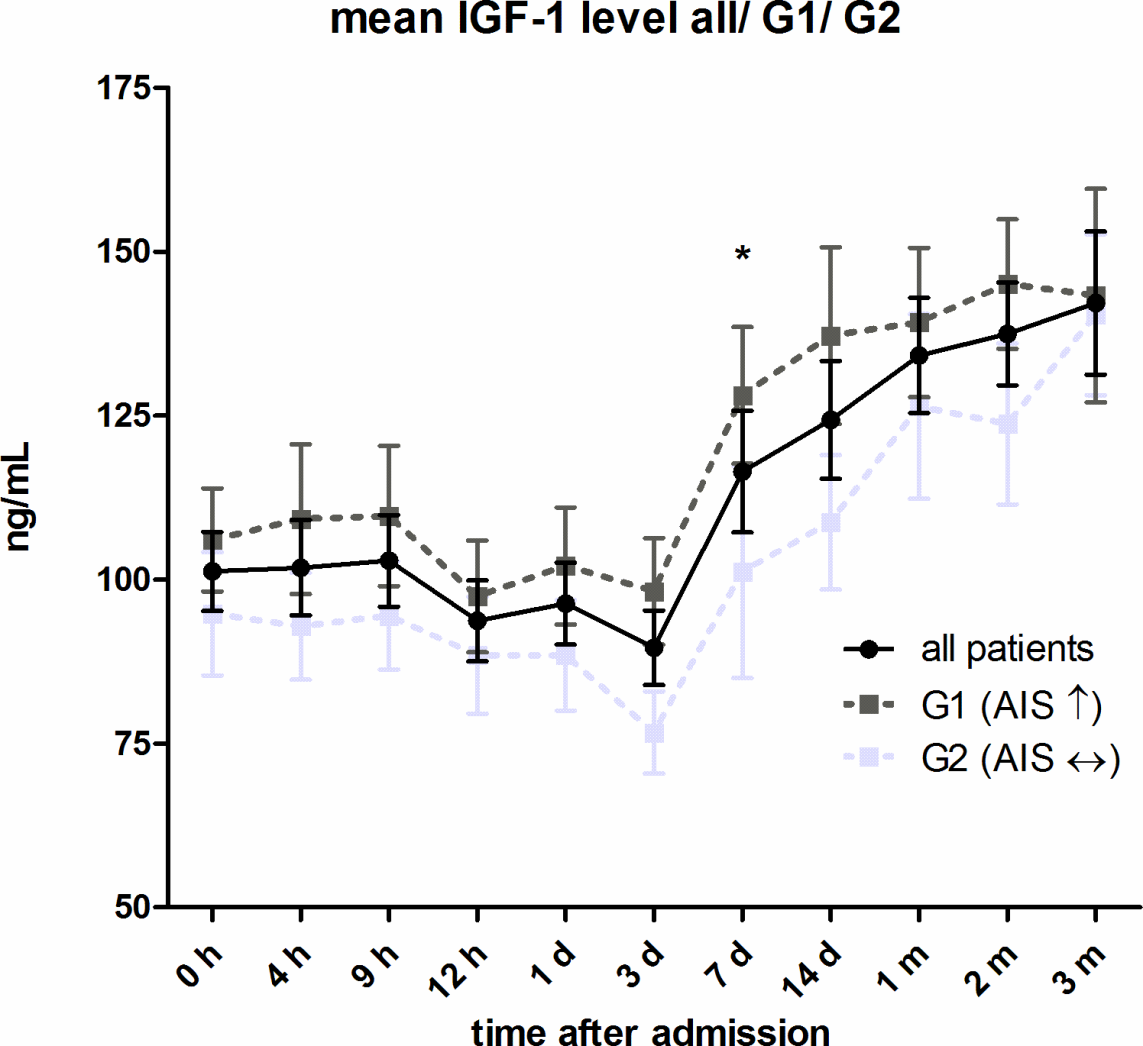
Mean IGF-1 serum level comparison of all patients with (G1) and without (G2) neurological remission. Expressed as mean values ± standard error of the mean. The Mann-Whitney-U-Test assessed significant differences between both groups at each particular time-point, *p < 0.05. For better illustration mean values of all traumatic SCI patients had been added to the figure.

(see Fig 3) The subgroups G1 and G2 matched in their profile for the entire collective. There was a slight decrease after 12 hours and levels increased 7 days after injury. However, G1 showed continuously higher IGF-1 levels than G2 from the beginning of the observation period until the end. The difference in IGF-1 levels between the two groups was significant 7d after trauma (p < 0.05; Mann-Whitney-U Test).

#### Comparison of all AIS A and all AIS B-D

**Fig 4:**
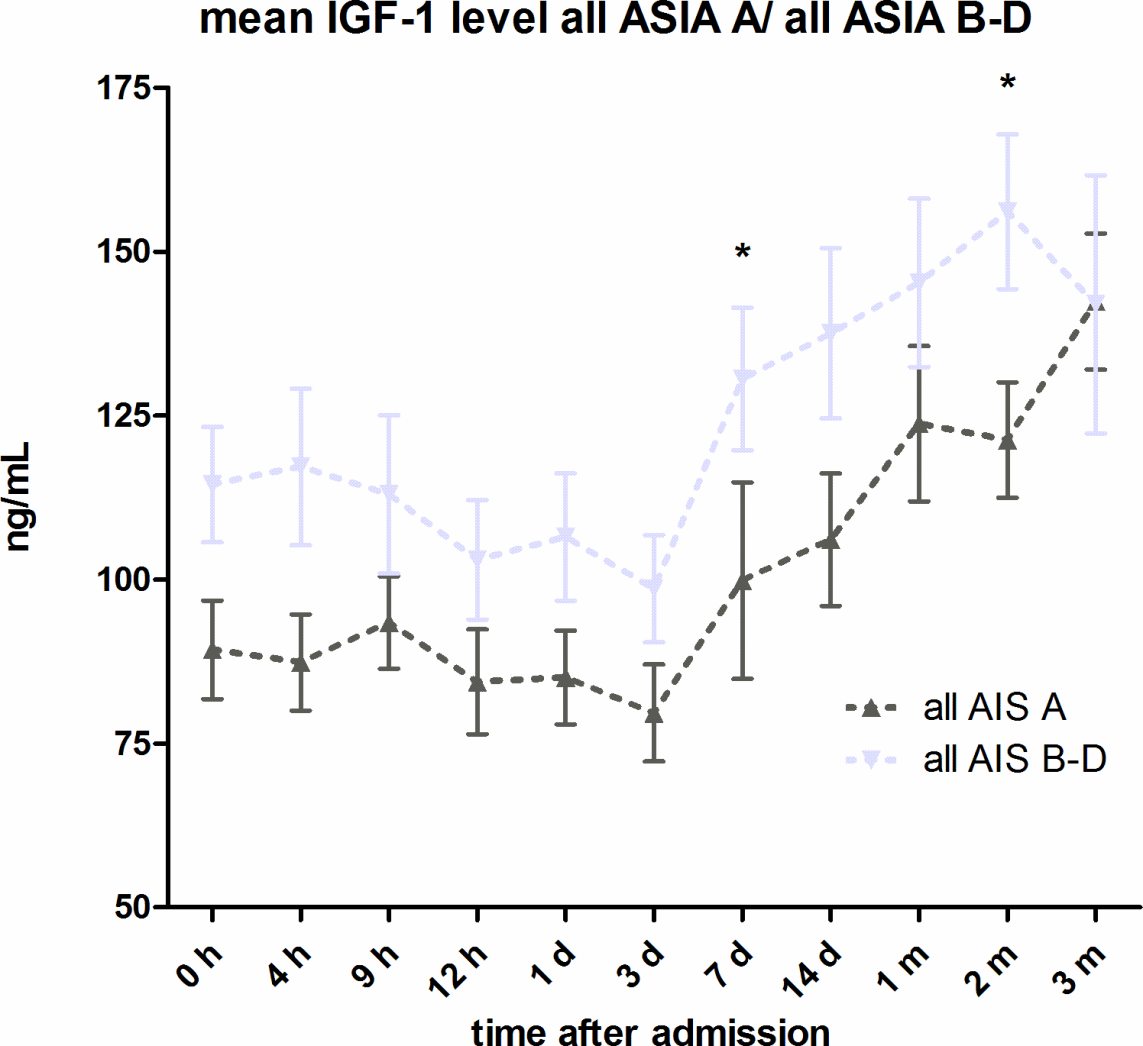
**IGF-1 serum level comparison of all AIS A classified patients and all AIS B-D classified patients.** Expressed as mean values ± standard error of the mean. The Mann-Whitney-U-Test assessed significant differences between both groups at each particular time-point, *p < 0.05.

(see Fig 4) During the twelve-week observation period, all initially AIS A classified patients showed constantly higher IGF-1 levels than initially AIS B-D classified patients, on average 22.86pg/mL (18.66%) higher. Measurements were significantly different seven days and two months after injury (p < 0.05; Mann-Whitney-U Test).

#### Location shifts between IGF-1 levels of 3 to 7 days after injury within All Patients, G1 and G2

**Fig 5:**
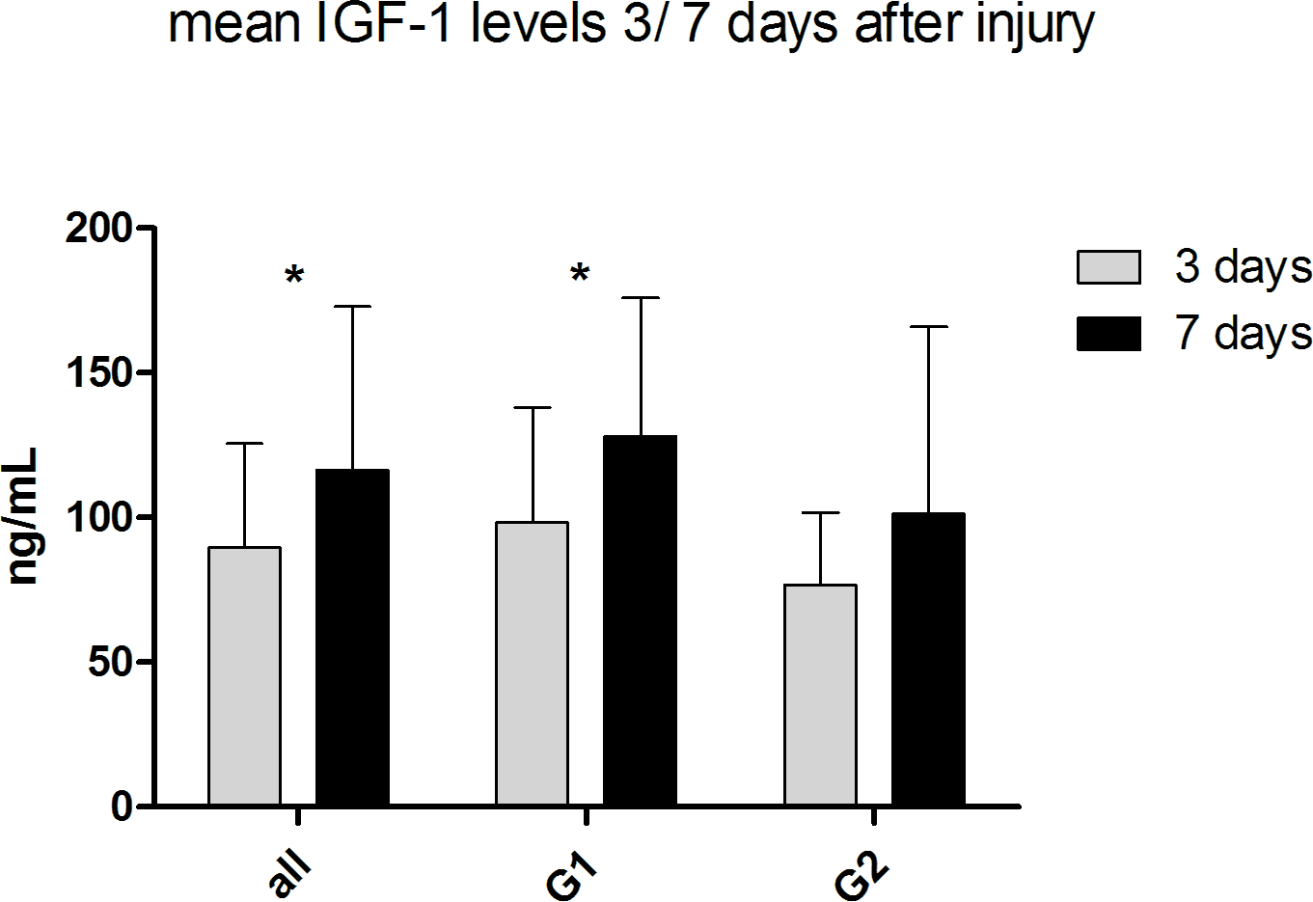
Location shifts between IGF-1 levels of 3 to 7 days after injury within all patients, G1 and G2. IGF-1 serum levels are expressed as mean values ± standard error of the mean. The Wilcoxon signed-rank test assessed significant differences from the IGF-1 level 3 days to 7 days after injury, *p < 0.05.

(see Fig 5) By analyzing only location shifts between IGF-1 levels of three to seven days after injury we found significant values in the group of all patients and G1 (G1: *p* = 0.01; G2: *p* = 0.012; Wilcoxon signed-rank Test). G2 (*p* = 0.079; Wilcoxon signed-rank Test) showed no significant difference.

All mean IGF-1 levels of all subpopulations are shown in S2 Fig. For a more detailed comparison of the major subpopulations (all patients/G1/G2/all AIS A/all AIS B-D) at each individual time-point see S3 Fig.

### Correlation

In the current study, we found a high correlation between the initial AIS score and the type of paralysis (*r = - 0*.*515*, *p < 0*.*01)*, the neurological level of injury *(r = 0*.*326*, *p < 0*.*05)*, the final AIS score (*r = 0*.*847*, *p < 0*.*01)* as well as neurological improvement (*r = 0*.*555*, *p < 0*.*01)*. Furthermore, we were able to show a high correlation between neurological improvement and the type of paralysis. There was neither a correlation between fracture (yes, no) and the IGF-1 serum level (*p* > 0.20) nor between operation (no, quantity) and the IGF-1 serum level (*p* > 0.20), respectively.

#### Group comparison

The Kruskal-Wallis test for the type of paralysis and neurological outcome shows a significant difference between the types of paralysis regarding the neurological outcome (*p* = 0.01) and confirms the relevance of our classification.

#### Regression

The regression model has an area under the curve (AUC) of 0.81.8% [CI: 68.4%–95.1%] as compared to 59.5% [CI: 41.9%–77.1%] for IGF-1 alone. Considering both the model fit indices and clinical relevance, we decided to include paralysis, neurological level of injury, mean IGF-1 serum level of the first 24h, dichotomized initial AIS score and age in the multiple regression model. See Table 3.

#### ROC-Analysis

**Fig 6:**
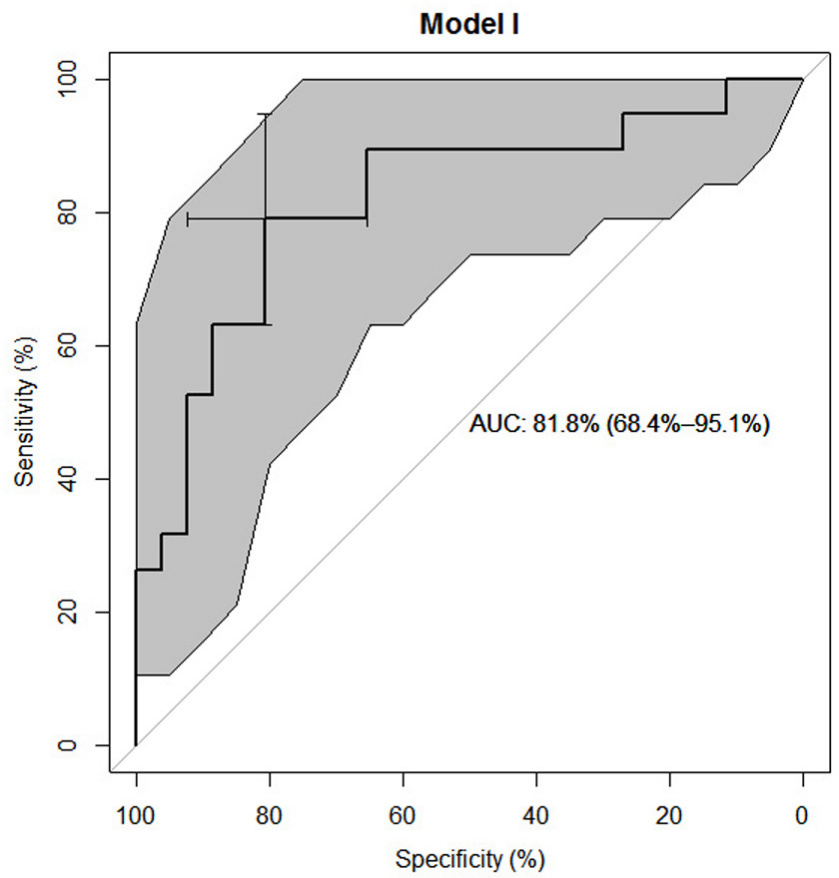
ROC Analysis of Model 1. Favored predictive model including all variables as predictors shown in Model 1 Table 3A (such as Paralysis: cP = complete Paraplegia, iC = incomplete Paraplegia, cT = complete Tetraplegia, iT = incomplete Tetraplegia; NLI = Neurological Level of Injury; MV = mean IGF-1 serum levels of the first 24 h; A_DB = dichotomized initial AIS = ASIA Impairment Scale, 0 = A, 1 = B-D; AGE = age of the subjects) which enables us to differentiate between neurological remission and no neurological remission in 81.8% [CI: 68.4%–95.1%] of cases. Grey highlighted area represents the particular confidence interval. Confidence interval of the model’s AUC in brackets. Model is based on *n* = 45 cases.

**Fig 7:**
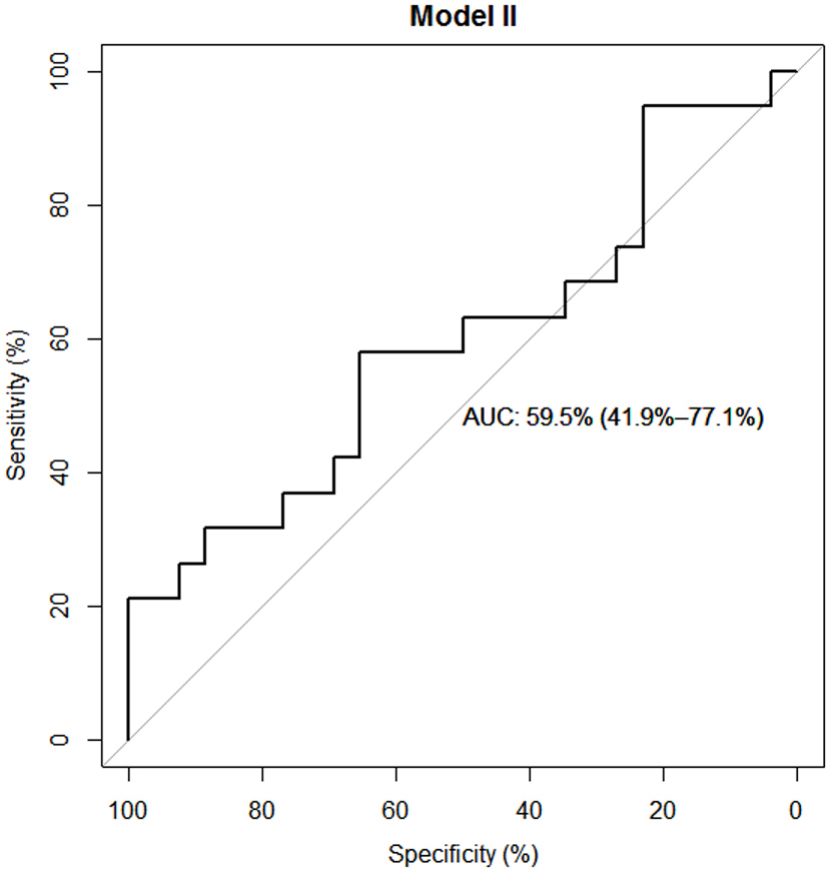
ROC Analysis of Model 2. Predictive model including the mean IGF-1 serum levels of the first 24h as predictor shown in Model 2 Table 3B. The model enables us to differentiate between neurological remission and no neurological remission in 59.5% [CI: 41.9%–77.1%] of cases. Confidence interval of the model’s AUC in brackets. Model is based on *n* = 45 cases.

ROC analyses were performed for the chosen multiple logistic regression model (AUC = 81.8% [CI: 68.4%–95.1%]; Model 1) as well as for the mean IGF-1 serum level of the first 24h (AUC = 59.5% [CI: 41.9%–77.1%], Model 2). See Figs 6 and 7.

## Discussion

One major finding of our prospective observational study is that IGF-1 shows a distinctive pattern in peripheral blood serum after SCI, regardless of whether remission has taken place. In the total collective, as well as in all subpopulations that were considered in this study, serum levels of IGF-1 increased 7 days after injury markedly above initial levels (see S2–S3 Figs and S1 Table). This result suggests that the expression of IGF-1 represents an important role during recovery after traumatic spinal cord injury. Furthermore, we could not estimate any correlation between fracture and/or operations and IGF-1 serum level (*p* > 0.20). Based on these results, we presume that changes in the IGF-1 serum level are directly related to neurological damage. The expression and release of IGF-1 is the peripheral main action and response to elevated serum levels of Growth Hormone (GH), also known as Somatotropin. GH binds to the extracellular domain of the transmembrane GH-Receptor (GH-R), which among others via intracellular binding of a tyrosine kinase, the Janus kinase 2 (JAK2), triggers a signaling cascade which ultimately leads to the expression of IGF-1 [26]. Signal Transducer and Activator of Transcription 5B (STAT5B) appears to be decisively involved in this process [27]. In addition to the JAK2-Stat pathway, the RAS/MAPK pathway, the PI3K/Akt pathway and its downstream partner, the mammalian (or mechanistic) target of rapamycin (mTOR) are considered gene transcription-triggering events [28–30]. In 2015 Y. Joshi et al described an additional way of signal transduction suggesting IGF1 signaling as a downstream effector of the MDM4 deletion. Following their observations, the MDM4/MDM2-p53-IGF1 axis is critical for axonal sprouting and neurological recovery after spinal cord injury. Furthermore, pharmacological enhancement of the MDM2/p53-IGF1R axis enhances axonal sprouting as well as functional recovery after spinal cord injury which might therefore be a new target in regenerative therapy [31]. Further studies revealed similar results. To examine post-regeneration events Bei et al performed a unilateral transection in the optic nerve of postnatal day 6 mice. Interestingly they found that co-overexpression of osteopontin (OPN)/IGF-1/ ciliary neurotrophic factor (CNTF) induces regrowth of retinal axons and formation of functional synapses in the superior colliculus and thus confirmed the results of Duan et al [32, 33]. IGF-1 changes its actions mostly by binding to one of the two IGF-Receptors, IGF-R1 and IGF-R2. These are anchored membrane-bound on the respective target cells and trigger various intracellular processes. Postnatal IGF-1 is especially involved in growth processes. Effects include the induction of the nutrients uptake, particularly amino acids and carbohydrates into muscle cells, as well as a fat loss effect by increased nitrogen retention. It also regulates bone turnover and the cartilage proliferation [18, 26]. Similar to findings in the axonal growth in the fetal Central Nervous System (CNS) [34, 35], Dupraz et al observed that axonal regeneration in adult CNS neurons requires re-expression and activation of IGF-1R and suggest that axon severing during disaggregation can trigger IGF-1R re-expression [36]. All these observations taken together suggest that the IGF-1/ IGF-1R pathway is a highly interesting and much promising target for therapy after nerve and spinal cord injury.

After severe trauma, the human body has to challenge an early catabolic flow due to hyper-metabolism [37–39]. IGF-1 might be able to improve protein anabolism already in an early stage of severe trauma and might therefore improve the recovery process and the patient’s outcome [40]. Through the induction of cryogenic lesions in the spinal cords of adult rats, Yao et al were able to show increasing levels of IGF-1 mRNA and peptide between three and seven days after injury, expressed in astrocytes [41]. IGF-1 levels continued to rise and reached their maximum after 21–28 days. Further studies showed similar time-points of increasing IGF-1 levels after experimentally induced spinal cord demyelination [42]. They concluded that early astrocytic production of IGF-I might be involved in myelin regeneration. Improved myelin regeneration is very much likely to have a positive effect on neuroprotection as myelin has important protective and nutritional functions for the nerve. In addition, myelination seems to be a limiting step toward functional recovery after axon regeneration [32]. Another study on IGF-1 was able to show retinal neovascularization after intravitreal injection of IGF-1 [43]. The angiogenic potential of IGF-1 could help re-establish blood flow to the lesioned area and therefore promote metabolism and regeneration and helps prevent ischemic damage. All the aforesaid mechanisms of action of IGF-1 would contribute in this sensitive early stage after injury to improve the supply of much needed nutrients and to accelerate the removal of cell-damaging substances from neurons and the intercellular space. With our data we have shown that it is possible to determine IGF-1 levels in peripheral venous blood serum and depict distinctive pattern.

Mean IGF-1 serum levels in G1 compared to G2 were significantly different 7 days after injury (p < 0.05). See Fig 3. By comparing all AIS A classified patients with all AIS B-D classified patients we found consistently higher IGF-1 levels in the group AIS B-D, significantly different seven days and two months after injury (p < 0.05). See Fig 4. On average, IGF-1 mean levels were 18.66% (22.86ng/mL) higher in AIS B-D compared to AIS A. AIS A is defined as no sensory or motor function preserved in the sacral segments 4–5 as a sign of a total loss of spinal cord connection to superordinate central nervous areas [44]. A grade of AIS B-D indicates incomplete motor and or sensory loss of function and is a sign of residual and unaffected tracts within the spinal cord. While this classification describes preserved function, it can be used to roughly estimate the severity of injury within the spinal cord. In general, AIS A classified patients show the most severe damage to the spinal cord and AIS B-D with decreasing seriousness [45–48]. In contrast, it is not always possible to conclude from the severity of the collateral damage to the bone and the soft tissue to the severity of the damage to the spinal cord [49]. As patients with neurological remission show consistently increased IGF-1 levels, we wanted to investigate whether the serum levels of the first 24 hours after trauma are already predictive for the outcome of the patients. From the multivariate analysis, we conclude that the predictive power of IGF-1 is low after adjusting for relevant co-variables. The clinical perspective suggests a mediation effect between the SCI level (predictor), the initial AIS score (mediator) and the neurological outcome (criterion). To verify our hypothesis, we compared two models: the final multiple logistic regression model described above and the same model without the dichotomized initial AIS score. In the reduced model, paralysis showed a much lower p value (p = 0.05 as compared to p = 0.19) which supports the hypothesis that the initial AIS score is indeed a mediator. However, the AIC of the reduced model deteriorated which suggests that the dichotomized initial AIS score adds additional predictive value over just using the SCI level. Regarding a predictive value mean, IGF-1 serum levels during the first 24 hours of observation are certainly irrelevant (adjusted Odd = 1.01, 95% CI .98 / 1.04) compared to the included covariates. Due to the sharp CI (see Table 3A, Model 1), enlargement of the study population provides no further information. The same applies for age. In contrast, the analysis of the dichotomized initial AIS score revealed a relevant adjusted Odd (= 0.16) and adjusted 95% CI 0.03 / 0.73. The neurological level of injury and the type of paralysis are not yet conclusive. Here, an enlargement of the study population would be necessary to determine their impact. As ROC curves for both the logistic regression model and the IGF-1 score are larger than 0.5 (IGF-1 only slightly), the regression model clearly shows a larger predictive value than IGF-1 alone. See Figs 6 and 7 for results.

After spinal cord injury, the body reacts most likely as a part of its repair mechanisms and thereby increased anabolic metabolism with an increased expression of IGF-1[40, 50, 51].This process can be understood in our study by the significant increase of IGF-1 levels in serum seven days after injury. A next step would be to establish an animal model to verify our results. To examine whether the peripheral to the central (at lesion side) administration of IGF-1 would result in different effects would therefore be of great interest. So far, there are no animal models which provide transferable results to humans in the field of traumatic spinal cord injury [16, 52]. In contrary some findings made in animal models e.g. *C*. *elegans* even provide opposite results. Byrne et al could demonstrate, that in aging animals Insulin/IGF1 signaling even inhibits age-dependent axon regeneration [53]. Again, this shows the difficult transferability of results from animal model experiments to humans and underlines the value of clinical trials. In a next step IGF-1 levels can be selectively raised in a suitable animal model. Therefore, our estimated time-points of the current protocol can be used as intervention times and observation times to correlate the results

## Conclusion

The neuroprotective effect of IGF-1 has already been suggested in previous works by other research groups. However, most of these studies were conducted in animal models, our aim was to investigate whether we could confirm these findings in human. In our study, we observed significant changes throughout the acute, subacute and intermediate phase after trauma in patients with spinal cord injury. All patients had increasing IGF-1 serum levels three days after trauma. The mean IGF-1 serum level was higher on average in patients with neurological remission. The consistency of the observed values suggests a possible influence of IGF-1 on neuroprotective processes. Our results lay the foundation for a new animal model which closely simulates human biochemical process after spinal cord injury and might facilitate the transfer of findings in animal models to humans.

## Supporting Information

S1 Fig Number of analyzed samples at each time-point during observation. Left Y-axis displaying the total number analyzed samples at each time-point, right Y-axis displaying the percentage of total enrolled patients (*n* = 45).
(TIF)

S2 Fig IGF-1 Serum levels of all subpopulations. All subpopulations considered during observation period of 3 months after injury, expressed as means values.
(TIF)

S3 Fig **comparison of IGF-1 serum levels of all patients/ G1/G2/ all AIS A/all AIS B-D at each individual time-point.** IGF-1 serum levels are displayed as boxplots and whiskers. Whiskers are displayed as 97,5 (upper whisker) and 2,5 (lower whiskers) confidence intervals. Dots are indicating outliers.
(TIF)

S1 Table IGF-1 serum levels of all subpopulations. All Patients, G1, G2, AIS A ↑, AIS A ↔, ASIA B-D ↑, AIS B-D ↔, all AIS A, all AIS B-D: Abbreviations: MV = mean value; SD = standard deviation; SME = standard mean error; MED = median; 1. IQR = 1. Interquartile range; 3. IQR = 3. Interquartile range; Min = minimum value; Max = maximum value.
AIS B-D: Only one sample could be analyzed for the time-point one month; for time-points two and three-month collection of samples failed and therefore no data could be analyzed.
(DOCX)

S2 Table Testing for Normality using KS-Test and Shapiro-Wilk-Test. The table is showing the test results for normal distribution of all measured IGF-1 serum level at each individual time-point. Grey-highlighted results represent all p < .05, indicating that the referring data is not normal distributed.
(DOCX)
