# Supplementary figures and images for: Elevated Serum Insulin-Like Growth Factor 1 Levels in Patients with Neurological Remission after Traumatic Spinal Cord Injury
Source: PLoS One. 2016 Jul 22;11(7):e0159764. doi: 10.1371/journal.pone.0159764 (PMC4957810; doi:10.1371/journal.pone.0159764)

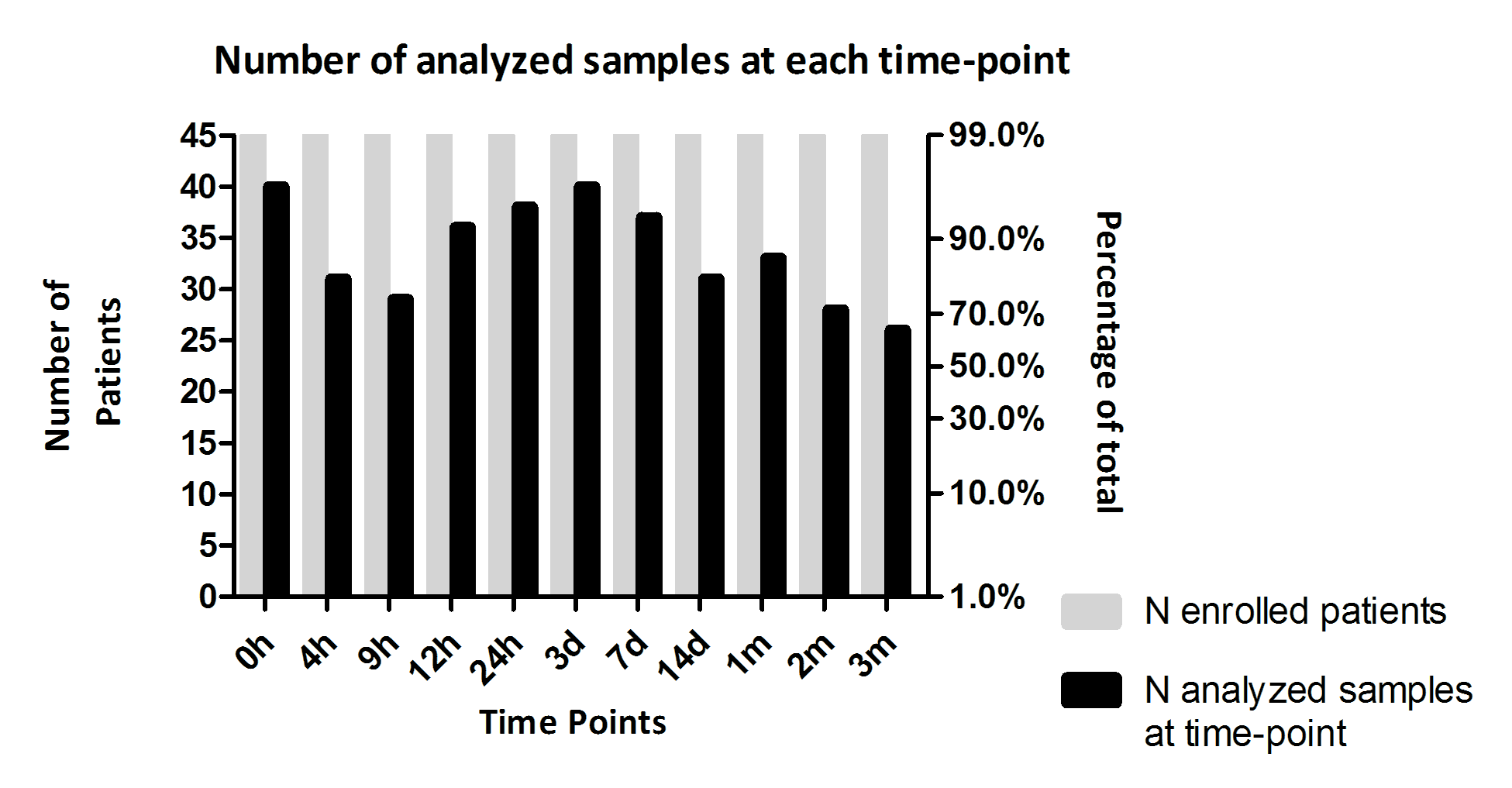

Supplement: S1 Fig — Left Y-axis displaying the total number analyzed samples at each time-point, right Y-axis displaying the percentage of total enrolled patients (n = 45). (TIF) [file pone.0159764.s001.tif]

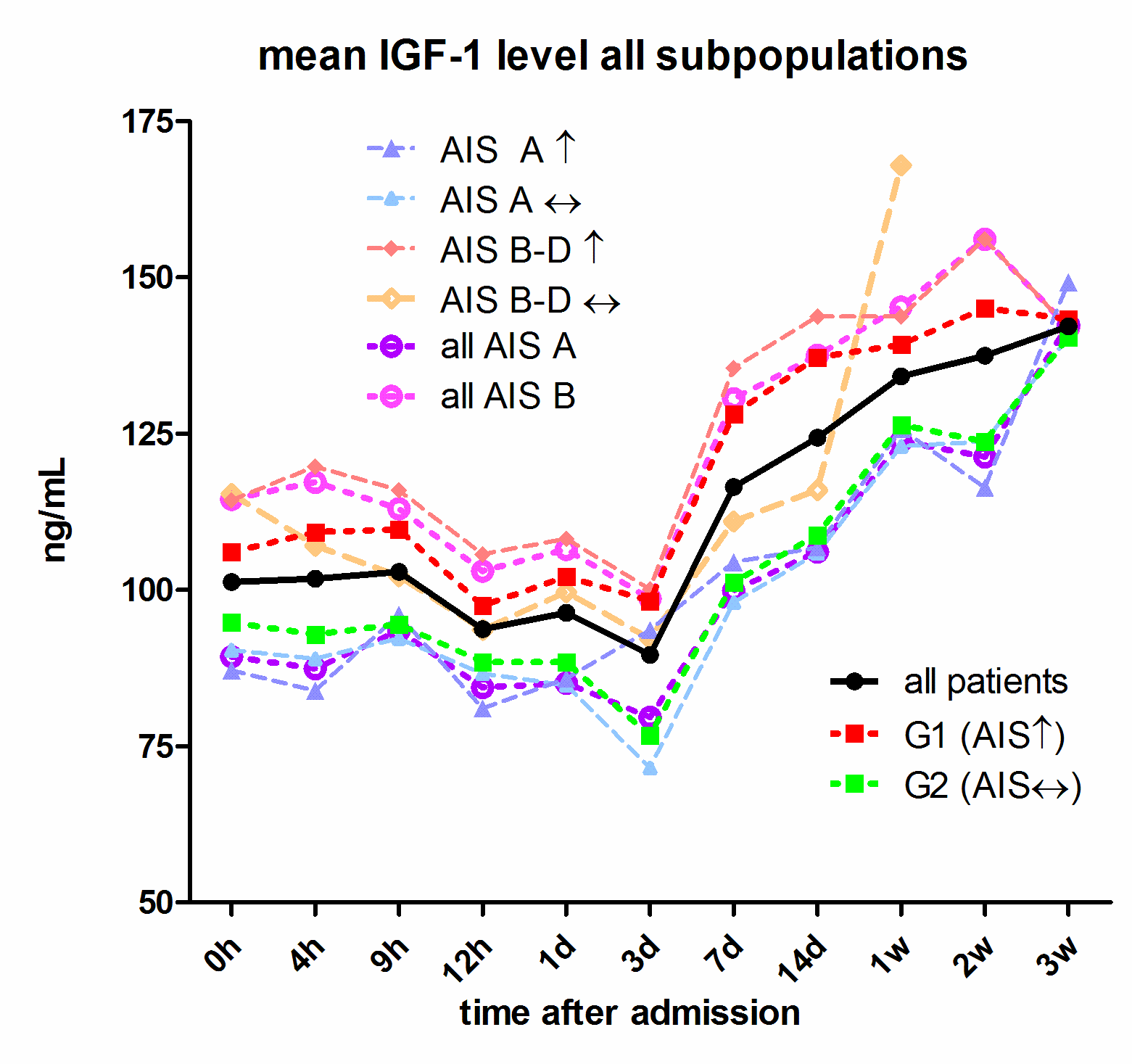

Supplement: S2 Fig — All subpopulations considered during observation period of 3 months after injury, expressed as means values. (TIF) [file pone.0159764.s002.tif]

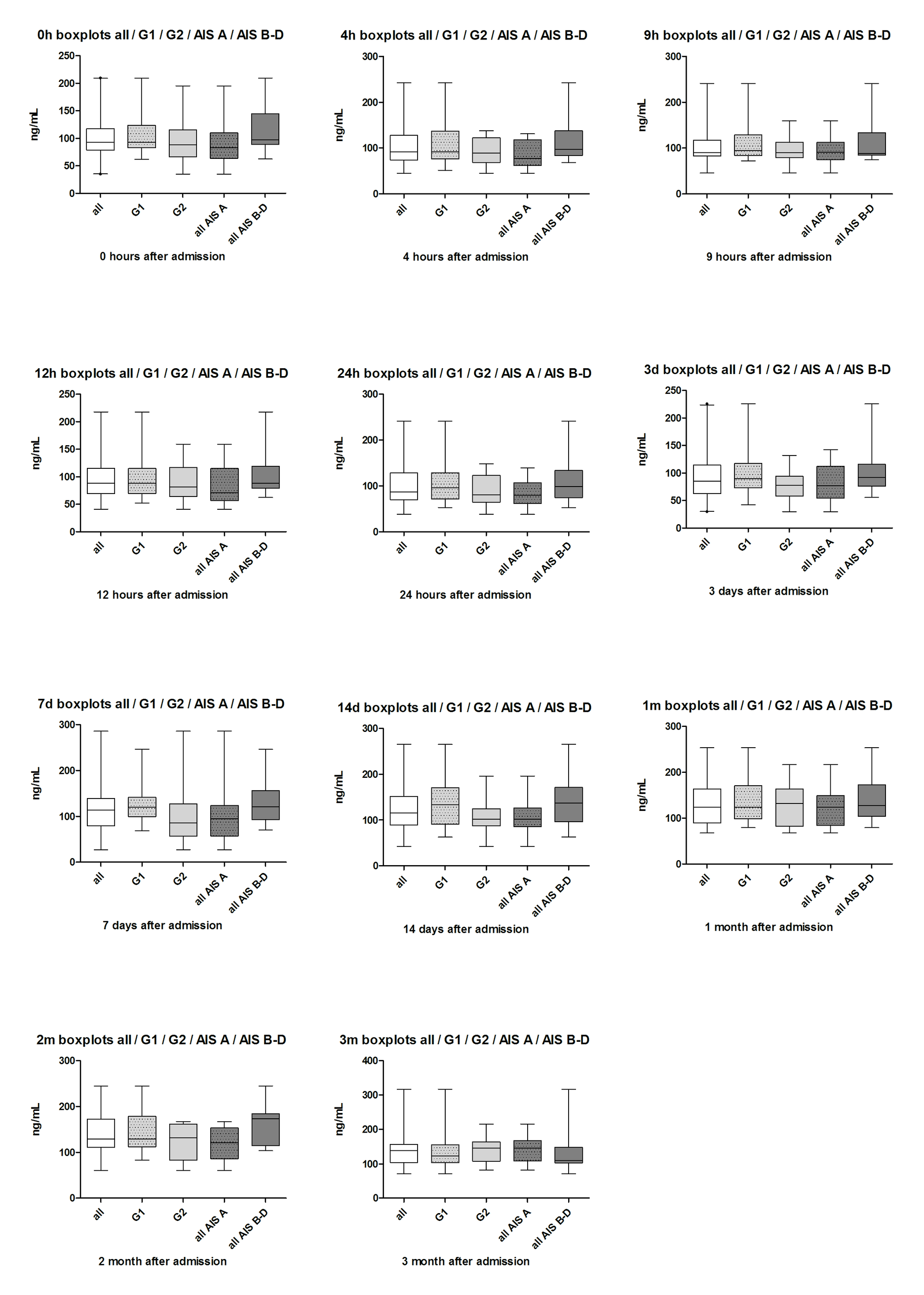

Supplement: S3 Fig — comparison of IGF-1 serum levels of all patients/ G1/G2/ all AIS A/all AIS B-D at each individual time-point. IGF-1 serum levels are displayed as boxplots and whiskers. Whiskers are displayed as 97,5 (upper whisker) and 2,5 (lower whiskers) confidence intervals. Dots are indicating outliers. (TIF) [file pone.0159764.s003.tif]
