# Supplementary material for: Elevated Serum Insulin-Like Growth Factor 1 Levels in Patients with Neurological Remission after Traumatic Spinal Cord Injury
Source: PLoS One. 2016 Jul 22;11(7):e0159764. doi: 10.1371/journal.pone.0159764 (PMC4957810; doi:10.1371/journal.pone.0159764)
Supplement: S1 Table — All Patients, G1, G2, AIS A ↑, AIS A ↔, ASIA B-D ↑, AIS B-D ↔, all AIS A, all AIS B-D: Abbreviations: MV = mean value; SD = standard deviation; SME = standard mean error; MED = median; 1. IQR = 1. Interquartile range; 3. IQR = 3. Interquartile range; Min = minimum value; Max = maximum value. AIS B-D: Only one sample could be analyzed for the time-point one month; for time-points two and three-month collection of samples failed and therefore no data could be analyzed. (DOCX) [file pone.0159764.s004.docx]

**S1 Table:** **IGF-1 serum levels of all subpopulations**. All Patients, G1, G2, AIS A ↑, AIS A ↔, ASIA B-D ↑, AIS B-D ↔, all AIS A, all AIS B-D: Abbreviations: MV = mean value; SD = standard deviation; SME = standard mean error; MED = median; 1. IQR = 1. Interquartile range; 3. IQR = 3. Interquartile range; Min = minimum value; Max = maximum value.

AIS B-D: Only one sample could be analyzed for the time-point one month; for time-points two and three-month collection of samples failed and therefore no data could be analyzed.

| All Patients (ng/mL)  n = 45 | | | | | | | | |
| --- | --- | --- | --- | --- | --- | --- | --- | --- |
| Time | MV | SD | SME | MED | 1. IQR | 3. IQR | Min | Max |
| 0h | 101,26 | 38,05 | 6,02 | 92,64 | 79,65 | 116,79 | 35,10 | 209,44 |
| 4h | 101,82 | 40,69 | 7,31 | 91,71 | 75,47 | 124,47 | 45,25 | 242,18 |
| 9h | 102,89 | 37,63 | 6,99 | 90,27 | 83,69 | 115,67 | 45,55 | 241,24 |
| 12h | 93,70 | 36,99 | 6,16 | 87,80 | 69,59 | 114,87 | 41,11 | 217,73 |
| 1d | 96,34 | 38,76 | 6,29 | 86,72 | 70,58 | 126,26 | 37,91 | 241,10 |
| 3d | 89,59 | 35,99 | 5,69 | 85,08 | 64,11 | 112,73 | 29,80 | 225,76 |
| 7d | 116,47 | 56,48 | 9,29 | 113,52 | 80,94 | 138,04 | 27,34 | 285,43 |
| 14d | 124,36 | 49,98 | 8,98 | 115,54 | 91,63 | 147,92 | 42,43 | 264,79 |
| 1m | 134,20 | 50,44 | 8,78 | 124,49 | 89,52 | 159,81 | 67,71 | 253,72 |
| 2m | 137,46 | 41,41 | 7,83 | 129,36 | 112,00 | 168,95 | 60,65 | 244,45 |
| 3m | 142,20 | 55,62 | 10,91 | 139,02 | 104,53 | 155,15 | 71,05 | 316,36 |

| G1 (ng/mL)  n = 26 | | | | | | | | |
| --- | --- | --- | --- | --- | --- | --- | --- | --- |
| Time | MV | SD | SME | MED | 1. IQR | 3. IQR | Min | Max |
| 0h | 106,04 | 37,70 | 7,86 | 92,84 | 84,34 | 120,78 | 62,21 | 209,44 |
| 4h | 109,19 | 47,13 | 11,43 | 91,71 | 78,75 | 136,42 | 51,65 | 242,18 |
| 9h | 109,70 | 42,85 | 10,71 | 94,70 | 84,30 | 121,82 | 72,30 | 241,24 |
| 12h | 97,46 | 39,05 | 8,52 | 87,93 | 69,68 | 114,72 | 52,30 | 217,73 |
| 1d | 102,07 | 41,92 | 8,94 | 95,68 | 72,97 | 126,26 | 53,16 | 241,10 |
| 3d | 98,19 | 39,92 | 8,15 | 89,77 | 74,77 | 116,70 | 42,54 | 225,76 |
| 7d | 128,11 | 47,75 | 10,42 | 120,10 | 101,56 | 139,98 | 68,65 | 245,75 |
| 14d | 137,21 | 55,66 | 13,50 | 133,38 | 103,72 | 168,48 | 62,80 | 264,79 |
| 1m | 139,25 | 50,85 | 11,37 | 124,31 | 100,40 | 163,42 | 79,45 | 253,71 |
| 2m | 145,08 | 41,85 | 9,86 | 129,36 | 112,67 | 177,53 | 83,23 | 244,45 |
| 3m | 143,32 | 65,20 | 16,30 | 123,12 | 103,68 | 153,33 | 71,05 | 316,36 |

| G2 (ng/mL)  n = 19 | | | | | | | | |
| --- | --- | --- | --- | --- | --- | --- | --- | --- |
| Time | MV | SD | SME | MED | 1. IQR | 3. IQR | Min | Max |
| 0h | 94,78 | 38,70 | 9,39 | 88,19 | 70,02 | 113,52 | 35,10 | 195,01 |
| 4h | 92,87 | 30,49 | 8,15 | 89,29 | 72,69 | 120,93 | 45,25 | 138,01 |
| 9h | 94,50 | 29,53 | 8,19 | 90,27 | 82,53 | 112,33 | 45,55 | 159,58 |
| 12h | 88,45 | 34,50 | 8,91 | 81,50 | 66,90 | 111,47 | 41,11 | 159,04 |
| 1d | 88,47 | 33,65 | 8,41 | 80,29 | 67,91 | 107,26 | 37,91 | 147,96 |
| 3d | 76,69 | 25,08 | 6,27 | 77,40 | 58,56 | 94,03 | 29,80 | 132,13 |
| 7d | 101,20 | 64,63 | 16,16 | 85,06 | 57,53 | 120,62 | 27,34 | 285,43 |
| 14d | 108,75 | 38,40 | 10,26 | 101,87 | 90,40 | 120,78 | 42,43 | 195,85 |
| 1m | 126,43 | 50,82 | 14,10 | 132,00 | 88,49 | 159,61 | 67,71 | 216,58 |
| 2m | 123,76 | 38,89 | 12,30 | 131,87 | 92,55 | 156,34 | 60,65 | 167,36 |
| 3m | 140,41 | 38,78 | 12,26 | 145,89 | 112,84 | 157,96 | 82,10 | 215,71 |

| all AIS A (ng/mL)  n = 23 | | | | | | | | |
| --- | --- | --- | --- | --- | --- | --- | --- | --- |
| Time | MV | SD | SME | MED | 1. IQR | 3. IQR | Min | Max |
| 0h | 89,29 | 34,39 | 7,51 | 82,78 | 64,72 | 107,01 | 35,10 | 195,01 |
| 4h | 87,39 | 29,38 | 7,35 | 77,92 | 66,40 | 115,37 | 45,25 | 131,20 |
| 9h | 93,46 | 27,28 | 7,04 | 90,74 | 78,85 | 108,79 | 45,55 | 159,58 |
| 12h | 84,41 | 33,79 | 7,97 | 70,75 | 59,40 | 112,09 | 41,11 | 159,04 |
| 1d | 85,09 | 30,28 | 7,14 | 80,22 | 64,01 | 99,37 | 37,91 | 139,36 |
| 3d | 79,64 | 32,30 | 7,41 | 76,57 | 56,21 | 103,05 | 29,80 | 142,30 |
| 7d | 99,86 | 61,79 | 14,99 | 94,49 | 57,83 | 117,28 | 27,34 | 285,43 |
| 14d | 106,08 | 36,42 | 10,10 | 102,07 | 89,17 | 123,27 | 42,43 | 195,85 |
| 1m | 123,79 | 48,80 | 11,84 | 124,14 | 86,58 | 138,81 | 67,71 | 216,58 |
| 2m | 121,30 | 34,11 | 8,81 | 120,77 | 98,80 | 149,81 | 60,65 | 167,36 |
| 3m | 142,43 | 37,47 | 10,39 | 146,01 | 110,24 | 161,12 | 82,10 | 215,71 |

| all AIS B-D (ng/mL)  n = 22 | | | | | | | | |
| --- | --- | --- | --- | --- | --- | --- | --- | --- |
| Time | MV | SD | SME | MED | 1. IQR | 3. IQR | Min | Max |
| 0h | 114,49 | 38,35 | 8,80 | 97,44 | 89,56 | 138,45 | 62,63 | 209,44 |
| 4h | 117,21 | 46,17 | 11,92 | 97,52 | 84,63 | 137,30 | 68,52 | 242,18 |
| 9h | 112,99 | 45,13 | 12,06 | 88,69 | 85,02 | 131,38 | 74,53 | 241,24 |
| 12h | 103,00 | 38,62 | 9,10 | 88,01 | 81,19 | 115,22 | 62,67 | 217,73 |
| 1d | 106,47 | 43,34 | 9,69 | 98,75 | 76,60 | 130,23 | 53,16 | 241,10 |
| 3d | 98,59 | 37,53 | 8,19 | 91,66 | 76,70 | 115,16 | 55,64 | 225,76 |
| 7d | 130,60 | 48,71 | 10,89 | 121,07 | 99,99 | 147,34 | 70,51 | 245,75 |
| 14d | 137,56 | 55,07 | 12,98 | 137,64 | 102,18 | 170,52 | 62,80 | 264,79 |
| 1m | 145,26 | 51,33 | 12,83 | 128,16 | 110,61 | 169,54 | 79,45 | 253,71 |
| 2m | 156,11 | 42,40 | 11,76 | 173,74 | 116,19 | 181,85 | 103,99 | 244,45 |
| 3m | 141,97 | 71,00 | 19,69 | 109,74 | 103,19 | 144,83 | 71,05 | 316,36 |

| AIS A ↑ (ng/mL)  n = 7 | | | | | | | | |
| --- | --- | --- | --- | --- | --- | --- | --- | --- |
| Time | MV | SD | SME | MED | 1. IQR | 3. IQR | Min | Max |
| 0h | 87,12 | 21,48 | 8,12 | 82,78 | 72,56 | 100,94 | 62,21 | 117,86 |
| 4h | 83,88 | 27,60 | 12,34 | 73,93 | 68,35 | 111,51 | 51,65 | 113,97 |
| 9h | 95,97 | 17,56 | 7,85 | 102,95 | 83,69 | 105,25 | 72,30 | 115,67 |
| 12h | 81,01 | 26,33 | 9,95 | 69,32 | 61,80 | 103,27 | 52,30 | 115,31 |
| 1d | 85,80 | 23,55 | 9,61 | 83,81 | 69,12 | 99,37 | 57,72 | 120,68 |
| 3d | 93,56 | 38,36 | 14,50 | 111,91 | 62,04 | 117,03 | 42,54 | 142,30 |
| 7d | 104,43 | 25,40 | 11,36 | 101,56 | 98,28 | 115,61 | 68,65 | 138,04 |
| 14d | 106,79 | 29,75 | 17,17 | 117,02 | 95,15 | 123,54 | 73,27 | 130,07 |
| 1m | 125,76 | 47,30 | 21,15 | 124,14 | 86,58 | 136,18 | 82,47 | 199,45 |
| 2m | 116,38 | 24,98 | 11,17 | 112,59 | 112,23 | 120,77 | 83,23 | 153,08 |
| 3m | 149,18 | 39,61 | 22,87 | 156,06 | 131,32 | 170,47 | 106,58 | 184,89 |

| AIS A ↔ (ng/mL)  n = 15 | | | | | | | | |
| --- | --- | --- | --- | --- | --- | --- | --- | --- |
| Time | MV | SD | SME | MED | 1. IQR | 3. IQR | Min | Max |
| 0h | 90,37 | 40,04 | 10,70 | 84,98 | 64,12 | 105,09 | 35,10 | 195,01 |
| 4h | 88,99 | 31,32 | 9,44 | 78,83 | 65,89 | 120,47 | 45,25 | 131,20 |
| 9h | 92,20 | 31,86 | 10,07 | 88,32 | 77,01 | 108,80 | 45,55 | 159,58 |
| 12h | 86,57 | 38,89 | 11,73 | 71,47 | 57,9 | 113,53 | 41,11 | 159,04 |
| 1d | 84,74 | 34,12 | 9,85 | 80,22 | 60,06 | 100,34 | 37,91 | 139,36 |
| 3d | 71,53 | 26,63 | 7,69 | 66,01 | 57,24 | 83,26 | 29,80 | 132,13 |
| 7d | 97,95 | 72,83 | 21,03 | 76,07 | 53,85 | 120,62 | 27,34 | 285,43 |
| 14d | 105,87 | 39,65 | 12,54 | 99,88 | 90,40 | 118,14 | 42,43 | 195,85 |
| 1m | 122,97 | 51,46 | 14,85 | 111,41 | 85,60 | 144,01 | 67,71 | 216,58 |
| 2m | 123,76 | 38,89 | 12,30 | 131,87 | 92,55 | 156,34 | 60,65 | 167,36 |
| 3m | 140,41 | 38,78 | 12,26 | 145,89 | 112,84 | 157,96 | 82,10 | 215,71 |

| AIS B-D ↑ (ng/mL)  n = 19 | | | | | | | | |
| --- | --- | --- | --- | --- | --- | --- | --- | --- |
| Time | MV | SD | SME | MED | 1. IQR | 3. IQR | Min | Max |
| 0h | 114,32 | 40,73 | 10,18 | 95,30 | 89,93 | 136,69 | 62,63 | 209,44 |
| 4h | 119,74 | 50,42 | 14,55 | 104,16 | 82,83 | 137,59 | 68,52 | 242,18 |
| 9h | 115,94 | 49,93 | 15,06 | 86,46 | 84,60 | 134,64 | 74,53 | 241,24 |
| 12h | 105,68 | 42,52 | 11,36 | 88,01 | 81,75 | 122,71 | 62,67 | 217,73 |
| 1d | 108,17 | 46,15 | 11,54 | 98,75 | 79,22 | 130,23 | 53,16 | 241,10 |
| 3d | 100,10 | 41,55 | 10,08 | 87,88 | 75,66 | 116,21 | 55,64 | 225,76 |
| 7d | 135,50 | 51,22 | 12,80 | 123,28 | 105,25 | 148,11 | 70,51 | 245,75 |
| 14d | 143,73 | 58,46 | 15,62 | 142,95 | 106,68 | 171,27 | 62,80 | 264,79 |
| 1m | 143,74 | 52,76 | 13,62 | 124,49 | 107,46 | 167,04 | 79,45 | 253,71 |
| 2m | 156,11 | 42,40 | 11,76 | 173,74 | 116,19 | 181,85 | 103,99 | 244,45 |
| 3m | 141,97 | 71,00 | 19,69 | 109,74 | 103,19 | 144,83 | 71,05 | 316,36 |

| AIS B-D ↔ (ng/mL)  n = 4 | | | | | | | | |
| --- | --- | --- | --- | --- | --- | --- | --- | --- |
| Time | MV | SD | SME | MED | 1. IQR | 3. IQR | Min | Max |
| 0h | 115,36 | 28,14 | 16,25 | 113,52 | 100,85 | 128,95 | 88,19 | 144,38 |
| 4h | 107,10 | 27,40 | 15,82 | 97,52 | 91,65 | 117,76 | 85,78 | 138,01 |
| 9h | 102,19 | 23,44 | 13,53 | 90,27 | 88,69 | 109,73 | 87,11 | 129,19 |
| 12h | 93,60 | 21,58 | 10,79 | 93,89 | 78,62 | 108,87 | 69,98 | 116,65 |
| 1d | 99,65 | 34,20 | 17,10 | 88,65 | 76,60 | 111,70 | 73,34 | 147,96 |
| 3d | 92,16 | 10,82 | 5,41 | 94,19 | 89,73 | 96,62 | 77,20 | 103,07 |
| 7d | 110,96 | 35,61 | 17,81 | 101,27 | 87,03 | 125,19 | 81,10 | 160,20 |
| 14d | 115,97 | 39,70 | 19,85 | 107,48 | 95,67 | 127,77 | 77,70 | 171,21 |
| 1m | 167,97 | - | - | 167,97 | 167,97 | 167,97 | 167,97 | 167,97 |
| 2m | - | - | - | - | - | - | - | - |
| 3m | - | - | - | - | - | - | - | - |
