# Supplementary material for: Elevated Serum Insulin-Like Growth Factor 1 Levels in Patients with Neurological Remission after Traumatic Spinal Cord Injury
Source: PLoS One. 2016 Jul 22;11(7):e0159764. doi: 10.1371/journal.pone.0159764 (PMC4957810; doi:10.1371/journal.pone.0159764)
Supplement: S2 Table — The table is showing the test results for normal distribution of all measured IGF-1 serum level at each individual time-point. Grey-highlighted results represent all p < .05, indicating that the referring data is not normal distributed. (DOCX) [file pone.0159764.s005.docx]

| Time-point | Cases | | | Kolmogorov-Smirnov  significance | Shapiro-Wilk  significance |
| --- | --- | --- | --- | --- | --- |
|  | Valid | Missing | total |  |  |
| At admission | 40 | 5 | 45 | 0,033 | 0,013 |
| 4 hours | 31 | 14 | 45 | 0,151 | 0,005 |
| 9 hours | 29 | 16 | 45 | 0,019 | 0,001 |
| 12 hours | 36 | 9 | 45 | 0,057 | 0,010 |
| 24 hours | 38 | 7 | 45 | 0,094 | 0,001 |
| 3 days | 40 | 5 | 45 | 0,031 | 0,004 |
| 7 days | 37 | 8 | 45 | 0,119 | 0,009 |
| 14 days | 31 | 14 | 45 | 0,200 | 0,133 |
| 1 month | 33 | 12 | 45 | 0,169 | 0,021 |
| 2 months | 28 | 17 | 45 | 0,200 | 0,550 |
| 3 months | 26 | 19 | 45 | 0,040 | 0,001 |

**S2 Table: Testing for Normality using KS-Test and Shapiro-Wilk-Test.** The table is showing the test results for normal distribution of all measured IGF-1 serum level at each individual time-point. Grey-highlighted results represent all p < .05, indicating that the referring data is not normal distributed.
